# Cranberry proanthocyanidins have anti-biofilm properties against *Pseudomonas aeruginosa*

**DOI:** 10.1186/1472-6882-14-499

**Published:** 2014-12-16

**Authors:** Robert K Ulrey, Stephanie M Barksdale, Weidong Zhou, Monique L van Hoek

**Affiliations:** Department of Biology, George Mason University, Manassas, Virginia USA; School of Systems Biology, George Mason University, Manassas, Virginia USA; Center for Applied Proteomics and Molecular Medicine, George Mason University, Manassas, Virginia USA; National Center for Biodefense and Infectious Diseases, George Mason University, Manassas, Virginia USA

**Keywords:** Cranberry, Proanthocyanidins, *Pseudomonas aeruginosa*, Biofilm

## Abstract

**Background:**

Bacteria within a biofilm are phenotypically more resistant to antibiotics, desiccation, and the host immune system, making it an important virulence factor for many microbes. Cranberry juice has long been used to prevent infections of the urinary tract, which are often related to biofilm formation. Recent studies have found that the A-type proanthocyanidins from cranberries have anti-biofilm properties against *Escherichia coli.*

**Methods:**

Using crystal violet biofilm staining, resazurin metabolism assays, and confocal imaging, we examined the ability of A-type proanthocyanidins (PACs) to disrupt the biofilm formation of *Pseudomonas aeruginosa*. We used mass spectrometry to analyze the proteomic effects of PAC treatment. We also performed synergy assays and *in vitro* and *in vivo* infections to determine whether PACs, alone and in combination with gentamicin, could contribute to the killing of *P. aeruginosa* and the survival of cell lines and *G. mellonella.*

**Results:**

Cranberry PACs reduced *P. aeruginosa* swarming motility. Cranberry PACs significantly disrupted the biofilm formation of *P. aeruginosa*. Proteomics analysis revealed significantly different proteins expressed following PAC treatment. In addition, we found that PACs potentiated the antibiotic activity of gentamicin in an *in vivo* model of infection using *G. mellonella*.

**Conclusions:**

Results suggest that A-type proanthocyanidins may be a useful therapeutic against the biofilm-mediated infections caused by *P. aeruginosa* and should be further tested.

**Electronic supplementary material:**

The online version of this article (doi:10.1186/1472-6882-14-499) contains supplementary material, which is available to authorized users.

## Background

Biofilms are colonies of bacteria encased within extracellular polymeric matrix [1]. Sessile biofilm bacteria are phenotypically different than planktonic bacteria, conferring increased resistance to desiccation, antibiotics, and the immune response. Antibiotics are able to kill the planktonic cells released by the biofilm after its maturation stages, but bacteria within the biofilm can persist, causing chronic infections [2]. In biofilm formation, bacteria attach reversibly to a surface, and then begin to produce extracellular polysaccharides. As the bacterial number grows, quorum sensing allows a phenotypic change in the bacteria. The biofilm matures and grows. Eventually, proteins break down parts of the matrix so that bacteria within the biofilm can disperse [2].

**Figure 1:**
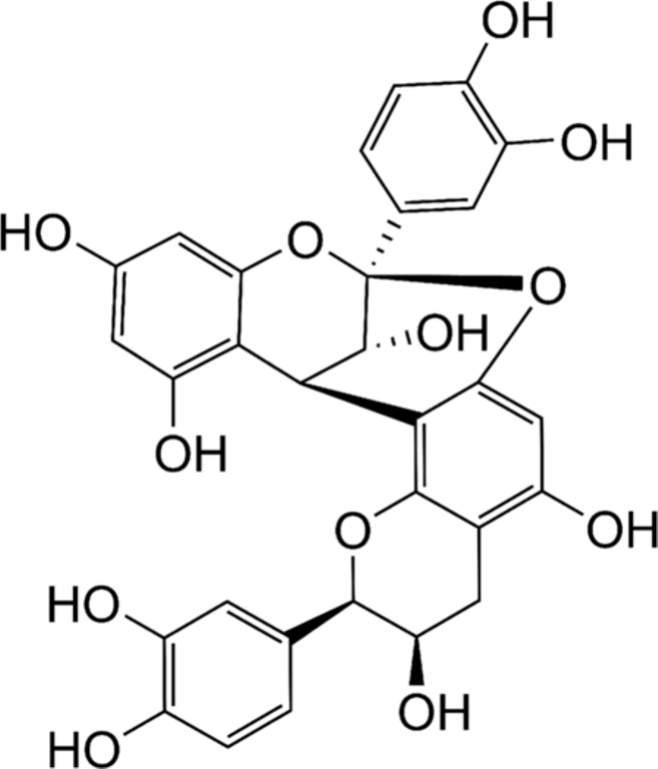
**Structure of the A-type proanthocyanidin monomer.** This structure represents the published chemical structure of PAC, the active ingredient in cranberry extract [8].

Cranberry juice has long been used to prevent infections of the urinary tract, which are often related to biofilm formation [3–5]. Recent studies have found that the A-type proanthocyanidins from cranberries have anti-biofilm properties against *Escherichia coli* [6, 7]. The primary active compound in cranberries is the condensed tannin A-type proanthocyanidins (PACs), which comprises about 65% of cranberry non-dialyzable material (NDM) [8]. This oligomer is comprised of several types of alpha-linked flavan-3-ols that are substituted variously with hydroxyls along the aromatic and fused oxytane rings, as shown in Figure 1. It has been found that A-type PACs are iron chelators, indicating that PACs may disrupt normal bacterial function by limiting the supply of iron [9]. PACs have been shown to prevent adhesion and reduce biofilm production by a variety of pathogens [10–14]. Research has revealed the ability of PACs to prevent the P-fimbriae adhesion of *Escherichia coli in vitro* and *in vivo* [15]. It has been shown that the alpha linkages in the compounds seem to be necessary to prevent adhesion [7]. Researchers have also found that PACs impair flagellum-mediated motility by *Pseudomonas aeruginosa* [16]. Thus, cranberry contains a variety of flavonols and proanthocyanidins that can be isolated and assessed for antimicrobial and anti-virulence activity [14, 17–22].

*P. aeruginosa* is a Gram negative, opportunistic human pathogen associated with colonization and infection of vital organs such as the lungs, urinary tract, and kidneys [23]. This is a serious issue for cystic fibrosis patients, for whom *P. aeruginosa* colonization in the lung is the leading cause of illness and death [24]. *P. aeruginosa* is associated with many hospital-acquired infections due to colonization of medical equipment. Infection is usually associated with sepsis and general inflammation. This bacterium is also implicated in the mixed biofilm infections of burn victims, chronic wounds and diabetic pressure ulcers [25]. Biofilm formation is a major virulence factor in *P. aeruginosa*, and treatments that address this element of its pathogenicity are greatly needed.

In these experiments, we studied the ability of cranberry PACs to inhibit biofilm formation of *P. aeruginosa,* potentiate the activity of gentamicin*,* as well as the ability of PACs to lessen *Pseudomonas* pathogenicity *in vitro* and *in vivo*. The identification of natural products that inhibit the biofilm formation of *P. aeruginosa* and other important pathogens of humans is of great interest.

## Methods

### Bacteria and materials

*P. aeruginosa* ATCC 9027 was obtained from the American Type Culture Collection (Manassas, VA). GFP-expressing *P. aeruginosa* (Strain: PAO1-pTDK-GFP) was a generous gift from Douglas Weibel from the University of Wisconsin (Madison, WI). Both strains were grown overnight in Tryptic Soy Broth (TSB, Difco 211825) in a shaking incubator at 37°C, bacterial pellets frozen in 20% glycerol at −80°C, and enumerated via serial dilution on Tryptic Soy Agar (Difco 236950). Cranberry PACs were generously provided by A. Howell (Rutgers University, NJ) and Ocean Spray.

### Swarming motility assay

An assessment of the swarming motility of *P. aeruginosa* strain PAO1 was performed as previously described with minor modification [26]. TSB alone or with 100 μg/mL PACs was inoculated with overnight grown bacterial stocks. Overnight growth was adjusted with sterile 1X PBS to OD_600_ 0.6. Petri dishes were filled with about 15 mL of modified M9 medium with 0.5% granulated agar (Difco 214530) and allowed to dry under laminar flow for 60 min. 5 μL of bacterial inoculum was placed in the center of the plate. Plates were incubated for 16 h at 30°C. Inhibition of swarming was measured qualitatively.

### Biofilm production inhibition assay

Biofilm inhibition by cranberry PACs was measured by the crystal violet stain method with some alterations [27]. *P. aeruginosa* ATCC 9027 (10^5^ CFU) in TSB was incubated with varying concentrations of PACs, or without treatment, in triplicate in a 96-well plate (Corning 353072) (24 h; 37°C). Initial turbidity was measured at OD_600_. Wells were washed with tap water, and the biofilm fixed at 60°C in a hybridization oven for 1 hour. The plate was stained with 0.1% crystal violet for 5 minutes, washed, and reconstituted with 33% acetic acid. The plate was read on a spectrophotometer at OD_590_. Student’s *t-test* was performed between the growth/biofilm ratio for each experimental value and the negative control, as determined by absorbance at OD_600_ and OD_590_.

### Disruption of pre-formed biofilm

The disruption of pre-formed biofilm by cranberry PACs was assayed using *P. aeruginosa* ATCC 9027 as previously described [25, 28]. After biofilm formation (24 h, 37°C) in a 96-well plate (Corning, 353072), various concentrations of PACs were added to the wells in triplicate. Wells without treatment served as the negative control. After an additional incubation (24 h, 37°C), bacterial growth was measured at OD_600_, and the crystal violet stain was performed as above in the biofilm production inhibition assay.

### Bacterial attachment assay

Attachment assays were performed in a 96-well microtiter plate (Corning, 353072) as previously described [25, 28]. Overnight cultures of *P. aeruginosa* ATCC 9027 were grown in TSB to an OD_600_ of ~1.0. Bacterial culture was added to wells containing varying concentrations of PACs, or no PACs in triplicate. The plates were incubated (1 h, 37°C) to allow attachment and the absorbance was measured at OD_600_. The crystal violet stain was performed as for the biofilm production assay [29].

### MIC and checkerboard assays

The minimal inhibitory concentration of gentamicin for *P. aeruginosa* ATCC 9027 was determined as previously in Mueller Hinton Broth [30]. A checkerboard assay was performed as previously described [31, 32].

### Confocal imaging of flow cell slides

The flow chamber was set up and inoculated as previously described, with some modifications [33]. Frozen GFP^+^*P. aeruginosa* (2 × 10^9^ CFUs) was incubated for 1 h at 37°C in TSB and then was injected in a flow cell unit (FC 284 Dual Channel Transmission Flow Cell, BioSurface Technologies Corp.) with TSB and 100 μg/mL ampicillin. PACs (10 μg in 1 mL) were injected simultaneously with the bacteria. Untreated GFP+ *P. aeruginosa* was used as a control. Slides were imaged with a Nikon TE-2000 confocal using Z-stack. Graphics generated using Nikon EZ-C1 software. The experiment was performed in duplicate.

### Resazurin metabolism measurement assay

*P. aeruginosa* ATCC 9027 was grown in a 96-well plate (Corning 353072) and treated with PACs in various concentrations, in the same manner as the biofilm production inhibition assay. After 24 h growth, media was aspirated, and wells washed with 1X PBS. Resazurin (0.0064%) in TSB-C was added to each well. The OD_590_ was read kinetically in a spectrophotometer (30 min; 37°C). The quantitative difference in metabolic reduction of resazurin was compared at the 15 min time point between experimental and control wells with a Student’s *t-test*.

### Calgary device

The Calgary device (Innovatech) HTP MBEC assay was performed according to manufacturer’s instructions with small changes. Bacteria (10^5^ CFU/well) were exposed to varying concentrations of PACs in TSB in the Calgary device plate and incubated for 24 h at 37°C). Pegs on the plate lid were washed twice in fresh 96-well plates with sterile PBS and sealed thoroughly on a third plate containing PBS. This plate was sonicated for 30 min with the sealed plate sitting on the surface of a water-bath sonicator, and the resulting disrupted biofilm was plated in triplicate via serial dilution on TSA plates. Experiment was repeated 6 times. Experimental and control CFU counts were compared via Student’s *t-test*.

### Proteomic analysis

Treated and untreated *P. aeruginosa* was grown overnight in TSB at 37°C in a shaking incubator, with or without 100 μg/mL PACs. 1 mL was centrifuged to pellet (13,200 rpm, 10 min) and resuspended in 1 mL distilled water. Bacteria were lysed by repeated freeze-thaw cycles. Protein content was measured by the BCA protein assay (Pierce) and samples adjusted to equivalent protein concentrations with sterile water. 100 μg of protein were reduced by 10 mM dithiothreitol (DTT) for 30 minutes at 37°C, and then alkylated by 50 mM iodoacetamide for 20 min at room temperature. The proteins were digested by trypsin at 37°C for 6 h in a buffer containing ammonium bicarbonate (50 mM, pH 9) and 2 M urea. The digestion mixture was then acidified by adding glacial acetic acid to a final concentration of 2% and desalted by ZipTip (Millipore). The peptides were analyzed by high sensitive LC-MS/MS using an LTQ-Orbitrap mass spectrometer (Thermo Fisher) as previously described [34]. Tandem mass spectra collected by Xcalibur (version 2.0.2) were searched against the NCBI *P. aeruginosa* PAO1 protein database using SEQUEST (Bioworks software from ThermoFisher, version 3.3.1) with full tryptic cleavage constraints, static cysteine alkylation by iodoacetamide, and variable methionine oxidation. Mass tolerance for precursor ions was 5 ppm and mass tolerance for fragment ions was 0.25 Da. The SEQUEST search results of proteomics data were filtered by the criteria “Xcorr versus charge 1.9, 2.2, 3.0 for 1+, 2+, 3+ ions; ΔCn > 0.1; ranked top #1; probability of randomized identification of peptide < 0.01”. Confident peptide identifications were determined using these stringent filter criteria for database match scoring followed by manual evaluation of the results. The “false discovery rate (FDR)” was estimated by searching a combined forward-reversed database as described by Elias [35]. The SEQUEST search results were exported to spreadsheets and compared. Pathway classifications were done manually using the *Pseudomonas* genomic database (pseudomonas.org) and the KEGG Pathway module.

### Treatment of infected mammalian cells with PACs

J774A.1 mouse macrophage cells (ATCC TIB-67) and HEK293T/17 human kidney epithelial cells (ATCC CRL-11268) were acquired from the American Type Culture Collection (Manassas, VA), and were grown from a frozen stock with limited passaging. Cells were grown in Dulbecco’s Modified Eagle Medium (DMEM) with 10% Fetal Bovine Serum (FBS) and 1% penicillin/streptomycin. Cells were pelleted, washed once with PBS, pelleted again, and then resuspended in DMEM with 10% FBS. A tissue culture-treated 96-well plate (Corning 353072) was seeded with 5 × 10^4^ cells per well. *P. aeruginosa* ATCC 9027 at a multiplicity of infection (MOI) of 500 was added for 15 h. PACs (10 μg/mL) were added to experimental wells simultaneously with the bacteria. Non-infected cells were used to establish a spontaneous LDH release control. LDH release was measured kinetically for 15–17.5 hours using a spectrophotometer at OD_490_. The readings of treated wells were compared at the 15 h time-point with control wells using a Student’s *t-test*. Controls and experimental wells were set up in triplicate and the assay was performed 3 times.

### *In vivo*treatment of infected *Galleria mellonella*(wax moth larvae)

*G. mellonella* larvae (waxworms) were purchased from Vanderhorst Wholesale (St. Mary’s, OH) and used as a model of bacterial infection as previously described [25]. All injections were done into the first proleg unless the first proleg was deformed. The second injection was into the second proleg if 2 injections were performed. The waxworms weighed between 0.23-0.38 g. Tuberculin needles (0.5 mL) were used for injections. The waxworms were stored in plastic petri dishes at 37°C after injection and assessed for vital signs daily [25, 30, 36].

### Statistical analysis

The means and the standard deviation were calculated for the indicated experiments. The statistical analysis performed was Student’s *t-test* with a level of significance of p < 0.05.

## Results and discussion

### PACs reduced *P. aeruginosa*motility

**Figure 2:**
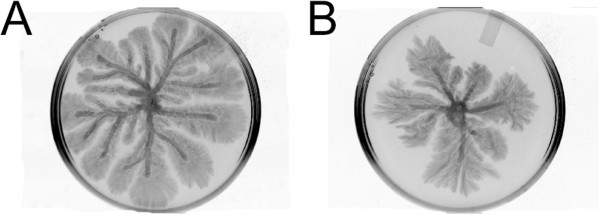
**Photographs of** ***P. aeruginosa*** **swarming motility on modified M9 agar, with A. modified M9 agar and B. modified M9 agar with 100 μg/mL cranberry PACs added.**

A previous study found that cranberry PACs inhibited the swarming motility of *P. aeruginosa* [16]. Our experiments confirmed that the swarming motility of *P. aeruginosa* was limited with the addition of 100 μg/mL PACs (Figure 2). Untreated bacteria swarmed to the edge of the agar plate (Figure 2A), while bacteria growing on PAC-containing agar moved about half the distance (Figure 2B). In addition, swarming patterns were not as branched or complex.

### Effect of cranberry PACs on *P. aeruginosa*biofilm

**Figure 3:**
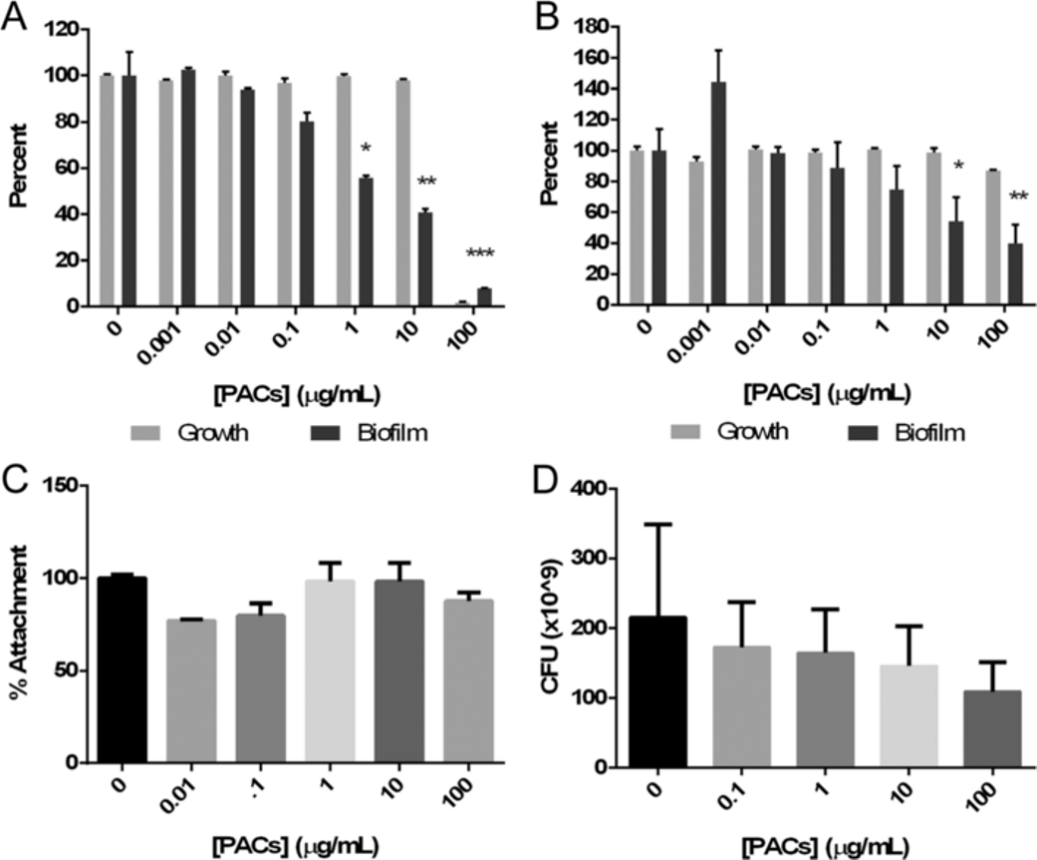
**Effects of cranberry PACs on** ***P. aeruginosa.*** **A**. biofilm formation (PACs dissolved in DMSO), **B**. pre-formed biofilm (PACs dissolved in deionized water), **C**. surface attachment, **D**. Bacterial number from treated and untreated biofilm using the Calgary device. * = p < 0.05; ** = p < 0.005; *** = p < 0.001.

Previous studies of both Gram-positive and Gram-negative bacteria have shown a significant decrease in biofilm production when exposed to cranberry juice, cranberry extracts, and cranberry PACs [10–14]. In our experiments, cranberry PACs significantly inhibited biofilm formation of *P. aeruginosa in vitro* at concentrations as low as 1 μg/mL (p < 0.05) (Figure 3A). At 1 μg/mL, PACs inhibited biofilm formation 40.9%, while 10 μg/mL PACs inhibited biofilm formation 55.7% (p < 0.01) compared to the untreated *P. aeruginosa*.

When bacteria were allowed to attach and form biofilm for 24 h before treatment, exposure to 10 μg/mL PACs for an additional 24 h resulted in a 54.1% (p < 0.05) reduction of preformed biofilm (compared to untreated control) and at 100 μg/mL, a 39.6% at (p < 0.01) inhibition compared to the untreated control (Figure 3B). PAC treatment showed no significant effect on the attachment of *P. aeruginosa* (Figure 3C). Using the Calgary device (consisting of inverted plastic pegs that hang down into the media of a 96 well plate, to which bacteria attach and form biofilm), exposure to PACs showed a 49.5% decrease in bacteria attached to the inverted pegs as compared to control at 100 μg/mL (p < 0.1), but no statistical difference at any other concentration (Figure 3D), confirming the preformed biofilm results above.

**Figure 4:**
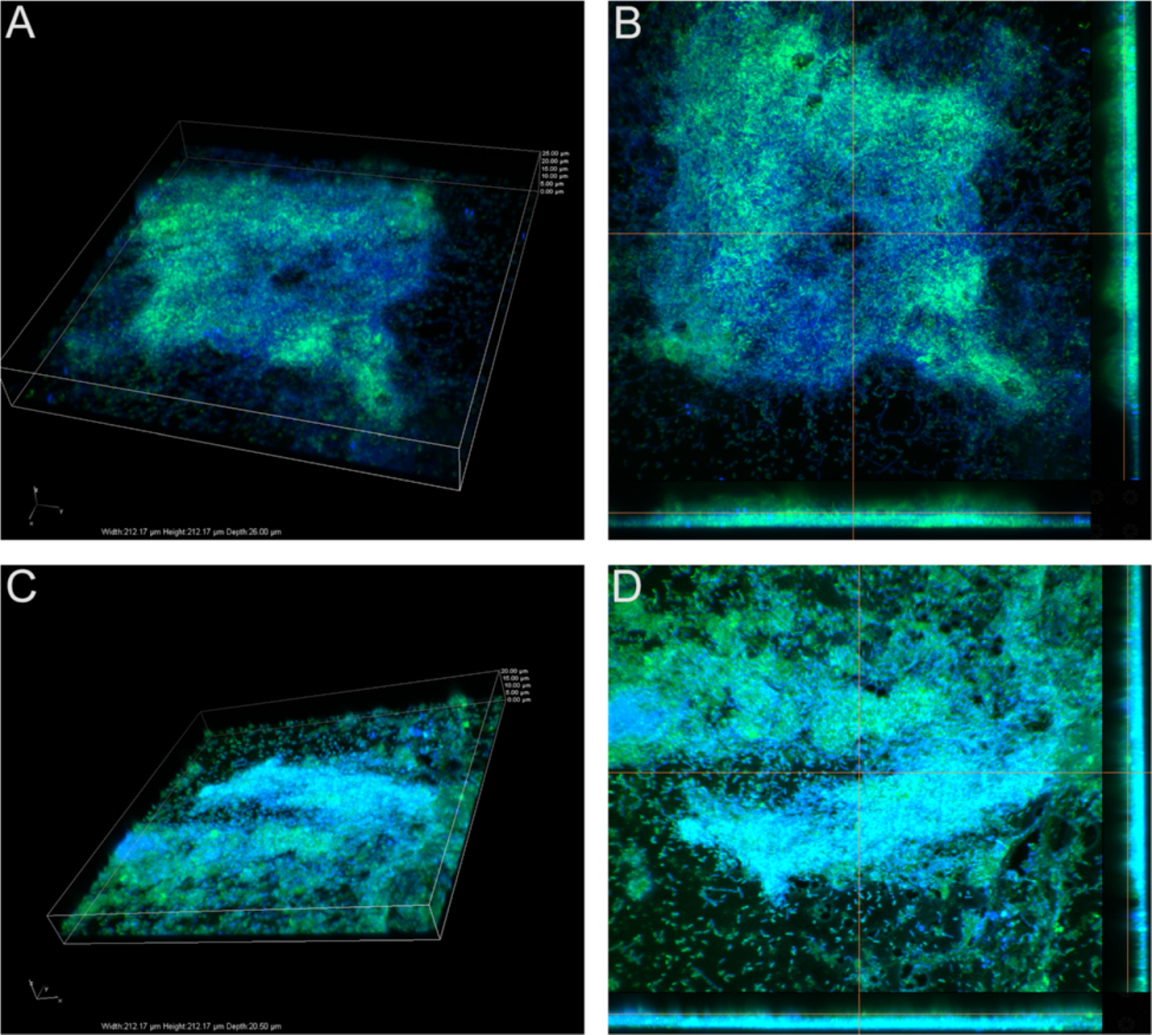
**Confocal imaging (3D Z stack and sections) of** ***P. aeruginosa*** **biofilm. A**. Untreated: 3-dimensional Z stack after 24 h incubation without PACs, **B**. Sections view after 24 h incubation without PACs, **C**. Treated: 3-dimensional Z stack after 24 h incubation with PACs, **D**. Sections view after 24 incubation with PACs.

In confocal imaging experiments in support of Figure 3A, imaged after the incubation of bacteria with PACs in a flow cell apparatus, it was found that biofilm height appeared to decrease after PAC treatment. Figures 4A - D illustrate the change in the height of *P. aeruginosa* biofilm, which decreased from ~26 μm to ~20 μm when treated with 10 μg PACs during attachment phase. In addition, the biofilm density also appeared to decrease.

### Proteomics of PAC-treated *P. aeruginosa*

**Figure 5:**
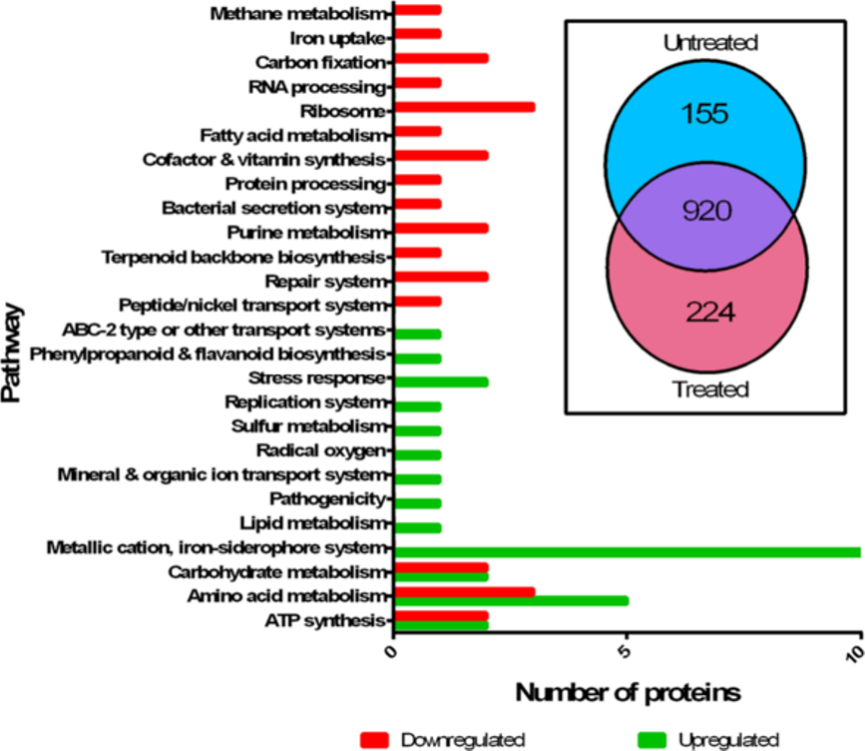
**A proteomics analysis of PAC treated** ***P. aeruginosa*** **.** The general function of the top 30 up-regulated and top 30 down-regulated proteins is illustrated, showing the general separation of function, and some overlapping functions between up- and down-regulated proteins. **Inset:** The number of proteins identified in the untreated and treated samples. Total of 1075 proteins were identified in untreated sample, and 1144 proteins were identified in treated samples. Total of 1299 proteins were identified in the two samples, and total of 920 common proteins were identified in both samples.

To understand changes in global protein expression of the bacterium in response to PACs treatment, *P. aeruginosa* was grown overnight with or without PACs (100 μg/mL), and then subjected to proteomic analysis by mass-spectrometry (LC-MS-MS) (3 replicates). A total of 1075 proteins were identified in the untreated sample, and 1144 proteins were identified in treated samples with 1% FDR (Figure 5A). The expression levels of the identified proteins in the two samples were compared by spectra count using the label-free quantitation method [34], and the result revealed many differentially expressed proteins (Additional file 1: Table S1).

Of 159 proteins that had the largest differences in abundance, the 30 most differentially regulated proteins were chosen from each group (up- and down-regulated) for examination in greater detail, as shown in Figure 5B. The top 30 proteins are listed in Table 1 (up-regulated) and Table 2 (down regulated). Proteins that were most significantly up-regulated (Table 1) include 12 proteins related to iron siderophores or cation transporters, for example, PchD, PvdN, and PhuS. 5 proteins putatively involved in amino acid synthesis were also up-regulated, including PA0335, PA2044, and HutG. In addition, several proteins that are up-regulated in response to stress were also found, such as OsmC and SodM. A hypothetical protein (PA3450) thought to be involved with flavonoid metabolism was also up-regulated.

**Table 1:** **The top 30 up-regulated proteins in PAC-treated** ***P. aeruginosa*** **bacteria**

Upregulated				
Proteins	Functional annotation	Accession number	Average spectra count	
			Untreated	Treated
fumarate hydratase FumC1	Central carbohydrate metabolism	15599666	0.1	21.3
ABC transporter ATP-binding protein PA4595	ABC-2 type or other transport systems	15599791	0.1	19
antioxidant protein PA3450	Phenylpropanoid and flavanoid biosynthesis	15598646	0.1	18
adhesion protein PA2407	Metallic cation, iron-siderophore and vitamin B12 transport system	15597603	0.1	17
3-oxo-C12-homoserine lactone acylase PvdQ	Metallic cation, iron-siderophore and vitamin B12 transport system	15597581	0.1	16
pyochelin biosynthesis protein PchD	Metallic cation, iron-siderophore and vitamin B12 transport system	15599424	0.1	15.3
hypothetical protein PA2410	Metallic cation, iron-siderophore and vitamin B12 transport system	15597606	0.1	13.3
hypothetical protein PA0335	Serine and threonine metabolism	15595532	0.1	12.3
protein PvdN	Metallic cation, iron-siderophore and vitamin B12 transport system	15597590	0.1	11.3
hypothetical protein PA4328	Stress protein	15599524	0.1	11
hypothetical protein PA3931	Metallic cation, iron-siderophore and vitamin B12 transport system	15599126	0.1	10
hypothetical protein PA4657	ATP synthesis	15599852	0.1	9.7
osmotically inducible protein OsmC	Stress protein	15595257	0.1	9
PmbA	Replication system	15599668	0.1	8.3
sulfite reductase CysI	Sulfur metabolism	15597035	0.1	7.3
superoxide dismutase SodM	Stress protein	15599664	0.1	6.7
hypothetical protein PA3250	Metallic cation, iron-siderophore and vitamin B12 transport system	15598446	0.1	6.7
hypothetical protein PA2699	Histidine metabolism	15597895	0.1	6.3
hypothetical protein PA2044	Cysteine and methionine metabolism	15597240	0.1	6.3
periplasmic polyamine binding protein PA0295	Mineral and organic ion transport system	15595492	0.1	6.3
heme/hemoglobin uptake outer membrane receptor PhuR	Metallic cation, iron-siderophore and vitamin B12 transport system	15599904	0.1	6
PhuS	Metallic cation, iron-siderophore and vitamin B12 transport system	15599903	0.1	5.7
pyocin S5	Pathogenicity	15596182	0.1	5.7
carbohydrate kinase PA3579	Lipid metabolism	15598775	0.1	5.3
hypothetical protein PA5229	Unknown function	15600422	0.1	5
heme uptake outer membrane receptor HasR	Metallic cation, iron-siderophore and vitamin B12 transport system	15598604	0.1	5
SpoVR family protein PA0586	Stress protein	15595783	0.1	5
hypothetical protein PA5359	Carbohydrate metabolism	15600552	0.1	4.7
N-formylglutamate amidohydrolase HutG	Histidine metabolism	15600284	0.1	4.3
amidotransferase PauD2	Histidine metabolism	15596939	0.1	4.3

**Table 2:** **The top 30 down-regulated proteins in PAC treated** ***P. aeruginosa*** **bacteria**

Downregulated				
Proteins	Functional annotation	Accession number	Average spectra count	
			Untreated	Treated
ABC transporter PA4502	Peptide/nickel export system	15599698	13.7	0
hypothetical protein PA2481	ATP synthesis	15597677	8	0
DNA topoisomerase I TopA	Repair system	15598207	7.7	0
homogentisate 1,2-dioxygenase HmgA	Aromatic amino acid metabolism	15597205	16.3	0.1
4-hydroxy-3-methylbut-2-en-1-yl diphosphate synthase GcpE	Terpenoid backbone biosynthesis	15598998	11.3	0.1
inosine 5′-monophosphate dehydrogenase GuaB	Purine metabolism	15598965	6.7	0.1
preprotein translocase subunit SecA	Bacterial secretion system	15599599	6.7	0.1
hypothetical protein PA5545	Protein processing	15600738	6.7	0.1
adenosylmethionine-8-amino-7-oxononanoate aminotransferase BioA	Cofactor and vitamin synthesis	15595617	6.3	0.1
hypothetical protein PA2765	Unknown function	15597961	6.3	0.1
3-ketoacyl-CoA thiolase FoaB	Fatty acid metabolism	15598209	6	0.1
acetyl-CoA carboxylase subunit beta AccD	Carbon fixation	15598308	5.7	0.1
30S ribosomal protein S3 RpsC	Ribosome	15599453	5.7	0.1
ribonuclease E Rne	RNA processing	15598172	5.3	0.1
bacterioferritin PA4880	Metallic cation, iron-siderophore and vitamin B12 xport system	15600073	5	0.1
50S ribosomal protein L13 RplM	Ribosome	15599629	4.7	0.1
formate dehydrogenase subunit epsilon FdhE	Other carbohydrate metabolism	15600003	4.7	0.1
hypothetical protein PA3967	Unknown function	15599162	4.3	0.1
lysine-specific pyridoxal 5′-phosphate-dependent carboxylase LdcA	Arginine and proline metabolism	15597015	4	0.1
acetyl-CoA carboxylase subunit A PA5436	Citrate cycle	15600629	4	0.1
cytochrome C PA2482	ATP synthesis	15597678	3.3	0.1
threonine dehydratase IlvA1	Branched-chain amino acid metabolism	15595528	3	0.1
transcription-repair coupling factor Mfd	Repair system	15598198	3	0.1
glycerate dehydrogenase HprA	Photorespiration	15599822	3	0.1
hypothetical protein PA5201	Pathogenicity	15600394	3	0.1
fumarase PA4333	Citrate cycle	15599529	41.3	1.7
phosphopantetheine adenylyltransferase CoaD	Cofactor and vitamin synthesis	15595560	14.3	0.7
aconitate hydratase PA0794	Citrate cycle	15595991	32.7	1.7
50S ribosomal protein L3 RplC	Ribosome	15599459	10.7	0.7
formyltetrahydrofolate deformylase PurU1	Purine metabolism	15599510	4.7	0.3

A wide variety of proteins were down-regulated due to PACs treatment (Table 2). This included 2 proteins related to ATP synthesis, a likely cytochrome C (PA2482) and hypothetical protein PA2481, and several proteins involved in DNA and RNA synthesis, such as TopA, RplC, and Mfd. In addition, several citric acid cycle proteins, such as subunits of the acetyl-CoA carboxylase and fumarase, were found to be significantly reduced.

Though it was found that the presence of PACs inhibited swarming motility, no proteins related to flagella or type IV pili were found be down-regulated. The quorum-sensing proteins in the *las* and *rhl* regulons were not found to be differentially regulated when treated, except for PvdQ, a quorum quencher [37], which had significantly more peptide hits with PACs treatment. It has been suggested that inhibition of quorum-sensing may be a mechanism to block *P. aeruginosa* biofilm production [37, 38].

### Further effect of cranberry PACs on biofilm-resident *P. aeruginosa*

**Figure 6:**
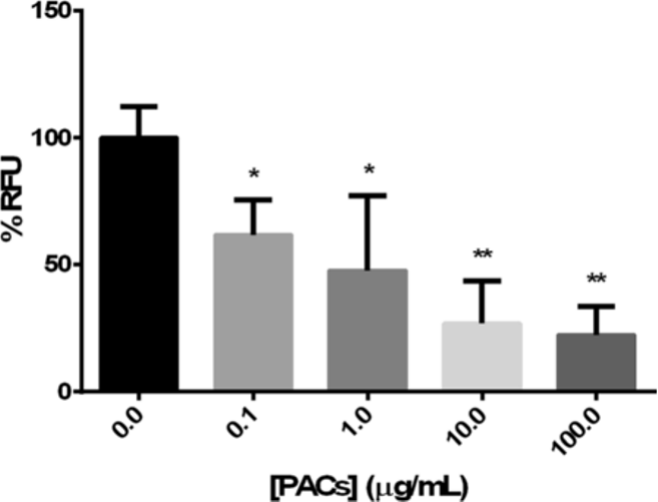
**Resazurin reduction of** ***P. aeruginosa*** **in biofilm following cranberry PAC treatment.** * = p < 0.05; ** = p < 0.005.

Metabolism in bacteria, as a measure of the number of bacteria present, can be measured with the compound resazurin, which becomes highly fluorescent when reduced to resorufin by chemicals or living cells, primarily by NADH oxidoreductases in bacteria. Biofilm-resident bacteria formed in the presence of higher concentrations of cranberry PACs had lower metabolic rates than biofilm-resident bacteria formed at lower concentrations of PACs (Figure 6). *P. aeruginosa* treated with 100 μg/mL PACs reduced 32.9% of the resazurin compared to the untreated bacteria (p < 0.01). At PAC concentrations of 10 μg/mL, PAC-treated *P. aeruginosa* biofilm reduced 36.9% of the untreated control (p < 0.01). Thus, these results most likely reflect a decrease in the total number of bacteria remaining in the PACs-reduced biofilm. Several citric acid cycle and ATP synthesis proteins were down-regulated, so the effect of PACs-treatment on *P. aeruginosa* biofilm may also be partly due to an effect on bacterial metabolism.

### *In vitro*potentiation of gentamicin by PACs

PAC is known to be an iron chelator [39], and some iron chelators have been shown to potentiate the activity of antibiotics, particularly aminoglycosides such as gentamicin and tobramycin [40]. To determine whether PACs may be able to contribute to potentiation of the antibiotic activity of gentamicin, a checkerboard assay was performed using varying concentrations of PAC against various concentrations of gentamicin. The PACs did not kill *P. aeruginosa* at any concentration tested, and thus are not directly antimicrobial. Gentamicin had an MIC of 1.5 μg/mL against this bacterial strain, which is in agreement with published values [41]. However, the MIC of gentamicin in combination with PACs was 1.3 μg/mL, as shown in Table 3. Based on these results, we concluded that PACs slightly potentiate the effect of the gentamicin *in vitro*. Cranberry PACs may be considered as an adjuvant to gentamicin.

**Table 3:** **Checkerboard assay of gentamicin and PACs against** ***P. aeruginosa,*** **showing the decrease (potentiation) of the gentamicin MIC by the addition of PAC**

	[Gentamicin] (μg/mL)								
[PAC] (μg/mL)		1.7	1.6	1.5	1.4	1.3	1.2	1.1	0
	100	0.081	0.084	0.079	0.088	0.178	0.698	0.841	1.74
	10	0.066	0.066	0.069	0.067	0.093	0.439	0.693	1.746
	1	0.057	0.055	0.054	0.059	0.07	0.461	1.137	1.743
	0.1	0.108	0.056	0.053	0.06	0.056	0.903	1.687	1.774
	0.01	0.056	0.054	0.063	0.053	**0.062**	1.002	1.197	1.794
	0	0.054	0.061	0.055	0.241	0.647	1.267	1.727	1.806

Bold value indicates point of synergy.

### Treatment of *P. aeruginosa-*infected J774A.1 and HEK293T/17 cells with PACs

**Figure 7:**
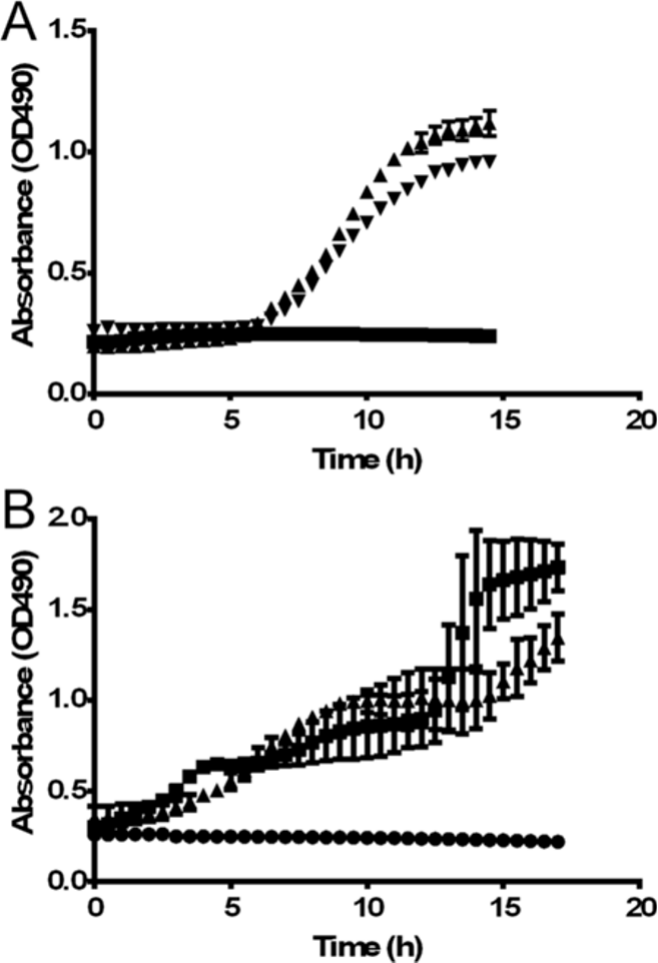
***In vitro*** **treatment of** ***P. aeruginosa*** **-infected mammalian cells with cranberry PACs. A**. J774A.1 mouse macrophage infected cell line. **B**. HEk293T/17 human kidney epithelial cell line. ▲ = untreated; ▼ = treated with PACs; ■ = no bacteria control.

In order to determine if PACs treatment could rescue eukaryotic cells during a *P. aeruginosa* infection, murine macrophage cells (J774A.1) and human embryonic kidney cells (HEK293T/17) were first treated with varying concentrations of PACs, and then infected with *P. aeruginosa* (MOI = 500) for 24 hours. The subsequent LDH release assay measured the release of lactate dehydrogenase, which is indicative of lysis. At 15 h, it was found that 10 μg/mL PACs significantly rescued murine macrophages from *P. aeruginosa*-mediated lysis, as shown by reduced release of LDH compared to the untreated control (p < 0.05, Figure 7A). This concentration of PACs also significantly rescued human embryonic kidney cells from lysis by *P. aeruginosa* (p < 0.05, Figure 7B)*,* with full kinetic data from the experiment shown.

### Treatment of *P. aeruginosa*-infected *G. mellonella*with cranberry PACs in combination with gentamicin

**Figure 8:**
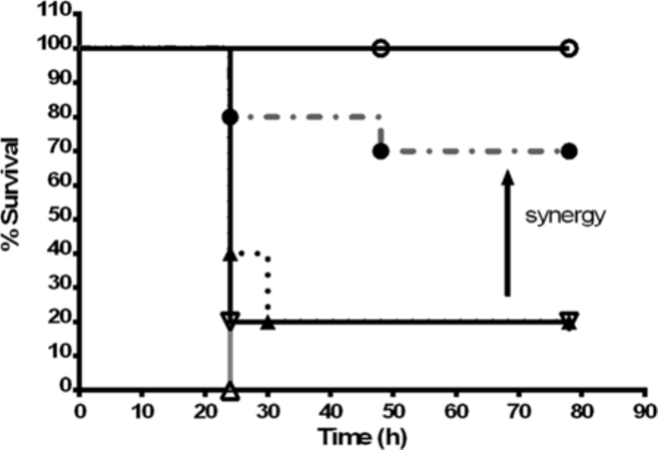
**PAC treatment of** ***P. aeruginosa*** **infection in** ***G. mellonella.*** Infected bacteria were exposed to PACs alone, gentamicin alone, or gentamicin and PACs together. ○ = 1 μg/mL PACs only; ∇ = 10^4^ CFU *P. aeruginosa*; ▼ = 10^4^ CFU *P. aeruginosa* treated w/ 0.5 μg/mL gentamicin; Δ = 10^4^ CFU *P. aeruginosa* treated w/ 1 μg/mL PACs; ● = 10^4^ CFU *P. aeruginosa* treated w/ 0.5 μg/mL gentamicin and 1 μg/mL PACs.

Some potentiating effect was shown (Table 3) when gentamicin was combined with PACs. To see if this effect would occur *in vivo*, *G. mellonella* was infected with *P. aeruginosa* and treated with sub-MIC concentrations of gentamicin and low levels of PACs. *G. mellonella* waxworms are an *in vivo* infection model for multiple pathogens, including *P. aeruginosa* [25, 30, 36]. A Kaplan-Meier survival analysis conducted on the results obtained over a 72 h period (Figure 8) indicated that a combination therapy of PACs and gentamicin was significantly (p < 0.05) more effective in reducing larvae death as compared to gentamicin treatment or PAC treatment alone. The average ratio of death over the 72 h time course between gentamicin alone, PAC alone, and gentamicin-PAC combination treatment was 3.4:1, suggesting the significant survival benefit of having the combination treatment.

## Conclusions

PACs are phenolic oligomers found in relatively high abundance within cranberries. Recent studies have found that these polyphenol structures are effective in reducing biofilm formation [42] *.* Cranberry juice and extracts have historically been used to prevent urinary tract infections in women [3–5]. The extracted NDM of cranberry has been shown to prevent the formation of biofilm by *S. epidermis* on soft contact lenses, which could reduce ocular infections [43]. It has been shown previously that cranberry NDM contains primarily A-type PACs [43]. Cranberry NDM has been shown to interfere with quorum sensing in *Vibrio harveyi* [44]. Cranberry NDM has also been shown to reduce the colonization of *Porphyromonas gingivalis* and mixed biofilms of *P. gingivalis* and *Fusobacterium nucleatum* in periodontal sites [45, 46].

In this study, we determined that cranberry PACs have anti-biofilm activities against the gram-negative bacterium *P. aeruginosa*. O’May and Tufenkji [47] showed that the sessile biofilm lifestyle of *P. aeruginosa* is bolstered by the presence of cranberry PACs, which limit motility, particularly swarming motility. Our experiments confirmed that PACs decreased the swarming motility of *P. aeruginosa,* both in the distance moved and the complexity of the swarming pattern.

We also found that PACs decreased biofilm produced by *P. aeruginosa* when applied continuously to the culture, which is consistent with published results in other Gram-negative bacteria such as *E. coli* and *P. gingivalis* [10–14]. Our experiments with cranberry PACs demonstrated a dose dependent reduction of preformed *P. aeruginosa* biofilm.

Several groups have found that P-fimbriae-mediated adherence to surfaces by *E. coli* is reduced by A-type PACs [6, 48]. Based on our attachment studies, PACs do not significantly reduce the adherence of *P. aeruginosa* to surfaces. *P. aeruginosa* attachment may not be affected due to the fact that this organism contains no P-fimbriae related attachment mechanisms. Planktonic growth is reduced by high concentrations of PACs, and PACs-treatment appears to reduce the total number of *P. aeruginosa* within the biofilm.

PACs have been shown to have an effect on a wide variety of bacterial systems, as their primary mechanism is iron chelation, an effect previously known in *E. coli* [9]. Proteomic analysis in this study shows that PAC treatment affects the abundance of many proteins in *P. aeruginosa*. Several of the proteins that are less abundant in the treated sample normally contain iron, such as cytochromes, suggesting a potential role of PAC iron chelation as a mechanism. Most notably, several of the up-regulated proteins are iron siderophores. In addition, Fe^3+^ is required for *P. aeruginosa* biofilms to fully mature into a large structure. It has been found that in iron-limiting conditions, *P. aeruginosa* will only form flat, thin biofilms [49], which we confirmed through fluorescent microscopy. Bacteria in these thin biofilms may be much less resistant to antibiotics and the host immune system than bacteria in a fully developed biofilm. Iron siderophores seem to be particularly effective as potentiators of aminoglycosides against *P. aeruginosa*, as it has been previously found that the activity of tobramycin was augmented when used in conjunction with lactoferrin [40].

We performed a series of experiments to explore how PACs may affect the action of a traditional antibiotic such as gentamicin. *In vitro* experiments showed the potentiation effect of PACs on the MIC of gentamicin. This was supported by *in vivo* studies in the *G. mellonella* model which demonstrated that a low dose of gentamicin with PACs prolonged survival in *P. aeruginosa*-infected worms significantly more than antibiotic or PACs alone. Thus cranberry PACs may be acting as antibiotic adjuvant for the action of gentamicin [50]. Cranberry juice has long been used as a prophylactic for urinary tract bacterial infection, and the use of PACs should be further explored to potentiate the action of antibiotics and reduce bacterial colonization and biofilm development during other infections caused by *P. aeruginosa*.

## Electronic supplementary material

Additional file 1: Table S1.: All up- and down-regulated proteins in PAC treated *P. aeruginosa* bacteria as determined by LC/MS/MS. (XLSX 101 KB)
